# A meta-analysis of internet-based cognitive behavioral therapy for military and veteran populations

**DOI:** 10.1186/s12888-023-04668-1

**Published:** 2023-04-03

**Authors:** Jenny JW Liu, Natalie Ein, Callista Forchuk, Sonya G. Wanklyn, Suriya Ragu, Samdarsh Saroya, Anthony Nazarov, J. Don Richardson

**Affiliations:** 1 The MacDonald Franklin OSI Research Centre, Parkwood Institute Research, Lawson Health Research Institute, Mental Health Building, 550 Wellington Road, RM F4-367, London, ON N6C 0A7 Canada; 2 Schulich School of Medicine, University of Western Ontario, London, ON Canada; 3 Operational Stress Injury Clinic, Parkwood lnstitute, Greater Toronto Area (GTA), ON Canada

**Keywords:** Cognitive-behavioral therapy, iCBT, Military, Veterans

## Abstract

**Background:**

Military and veteran populations are unique in their trauma exposures, rates of mental illness and comorbidities, and response to treatments. While reviews have suggested that internet-based Cognitive Behavioral Therapy (iCBT) can be useful for treating mental health conditions, the extent to which they may be appropriate for military and veteran populations remain unclear. The goals of the current meta-analysis are to: (1) substantiate the effects of iCBT for military and veteran populations, (2) evaluate its effectiveness compared to control conditions, and (3) examine potential factors that may influence their effectiveness.

**Methods:**

This review was completed following the Preferred Reporting Items for Systematic Reviews and Meta-Analyses (PRISMA) reporting and Cochrane review guidelines. The literature search was conducted using PsycInfo, Medline, Embase, and Proquest Dissertation & Theses on June 4, 2021 with no date restriction. Inclusion criteria included studies that: (1) were restricted to adult military or veteran populations, (2) incorporated iCBT as the primary treatment, and (3) evaluated mental health outcomes. Exclusion criteria included: (1) literature reviews, (2) qualitative studies, (3) study protocols, (4) studies that did not include a clinical/analogue population, and (5) studies with no measure of change on outcome variables. Two independent screeners reviewed studies for eligibility. Data was pooled and analyzed using random-effects and mixed-effects models. Study data information were extracted as the main outcomes, including study condition, sample size, and pre- and post-treatment means, standard deviations for all assessed outcomes, and target outcome. Predictor information were also extracted, and included demographics information, the types of outcomes measured, concurrent treatment, dropout rate, format, length, and delivery of intervention.

**Results:**

A total of 20 studies and 91 samples of data were included in the meta-analysis. The pooled effect size showed a small but meaningful effect for iCBT, *g* = 0.54, *SE* = 0.04, 95% *CI* (0.45, 0.62), *Z* = 12.32, *p* < .001. These effects were heterogenous across samples, (*I*^2^ = 87.96), *Q*(90) = 747.62, *p* < .001. Predictor analyses found length of intervention and concurrent treatment to influence study variance within sampled studies, *p* < .05. Evaluation of iCBT on primary outcomes indicated a small but meaningful effect for PTSD and depression, while effects of iCBT on secondary outcomes found similar results with depression, *p* < .001.

**Conclusions:**

Findings from the meta-analysis lend support for the use of iCBT with military and veteran populations. Conditions under which iCBT may be optimized are discussed.

**Supplementary Information:**

The online version contains supplementary material available at 10.1186/s12888-023-04668-1.

## Introduction

The COVID-19 pandemic has transformed mental health care, creating increased demand for remotely delivered services. Emerging research has noted a 154% increase in utilization of telehealth (i.e., reception of care without an in-person visit) since the pandemic, and underscored its utility during periods of in-person service closures and physical distancing mandates. [1, 2] Examinations of the impacts of telehealth have found these services to be effective for the general population. [3] Reviews suggest that telehealth delivery of psychological treatments may be particularly useful as a cost-effective alternative to in-person psychotherapy and to supplement access for rural communities. [4, 5] One particular population that could benefit from telehealth services is military and veteran populations.

### Evidence of effectiveness of iCBT

One type of telehealth service that has been promising is internet-based cognitive behavioral therapy (iCBT). Loosely described, iCBT involves delivery of cognitive behavioral therapy (CBT) through a computer, phone, or mobile device, often guided by a mental health professional. [6] iCBT has been demonstrated as effective in the treatment of mental health conditions, including posttraumatic stress disorder (PTSD) [8], depressive disorders [6, 9–11], anxiety disorders [6, 9, 11], substance use disorders [6], and insonmia [12] in the general population. Indeed, some have reported comparability between guided iCBT and in-person CBT. [6, 11]

While reviews generally agree that iCBT can be useful for treating mental health conditions, authors have highlighted several limitations that may affect interpretations of iCBT effectiveness and generalizability. First, variability in iCBT study design, such as allowance for concurrent treatments, length of intervention, presence of facilitation, delivery format, outcomes measured, and intended treatment targets may largely determine its overall effectiveness. Second, the degree to which iCBT may be tolerated by diverse patient populations remains unclear.

### iCBT for military and veteran populations

Veteran and active service military members are considered a vulnerable population on account of elevated rates of mental health concerns and physical and mental health service utilization. [13] Military and veteran communities are distinguished by uniqueness in frequencies and complexities of trauma exposures, which increases risk for the development of mental health conditions. [13, 14] Further, this population exhibits distinctions in symptom presentation and comorbidities [15], and is often less responsive [16], or differentially responsive [17] to mental health treatments compared to general populations. Interestingly, research has found iCBT to be an effective treatment for insomnia [18] and PTSD [7] within military and veteran populations. However, the variability in iCBT study design, as stated above, remain unclear among this population. Specifically, in reviewing available meta-analyses conducted on internet-based interventions, only four articles included, or focused on, military populations. [7–10] However, none of the meta-analyses focused specifically on iCBT within a military population or examined effects across various demographics, characteristics of iCBT interventions, and mental and physical outcomes.

### Aims and scope of review

In consideration of these factors, evaluations of iCBT for military and veteran populations is warranted. Specifically, pandemic-related restrictions and higher rates of mental health concerns coincide with increased telehealth treatment utilization. With the unique experiences and needs of military members and veterans, the effectiveness of iCBT for this population requires further exploration. To extend prior reviews, we sought to include diverse mental health outcomes and methodologically-divergent studies to examine factors that may differentially influence study effects. The goals of the current meta-analysis are to: (1) substantiate the effects of iCBT for military and veteran populations, (2) evaluate its effectiveness compared to control conditions (e.g., waitlist, treatment-as-usual [TAU], and active alternative interventions), and (3) examine potential factors that may influence the effectiveness of iCBT interventions, such as population characteristics, study design, and treatment delivery.

## Methods

### Search terms

This review was completed following the Preferred Reporting Items for Systematic Reviews and Meta-Analyses (PRISMA) [19] reporting and Cochrane [20] review guidelines. The literature search was conducted using four databases: PsycInfo, Medline, Embase, and Proquest Dissertation & Theses on June 4, 2021, with no date restriction. See supplementary S1 for search string.

### Inclusion and exclusion criteria

Our inclusion criteria included studies that: (1) were restricted to adult military or veteran populations, (2) incorporated iCBT (including computer, phone, mobile) as the primary treatment, and (3) evaluated mental health outcomes (e.g., PTSD, anxiety). Exclusion criteria included: (1) literature reviews, (2) qualitative studies, (3) study protocols, (4) studies that did not include a clinical or analogue population, and (5) studies with no measure of change on outcome variables (e.g., measure collected at pre-iCBT only).

### Study selection

**Fig. 1:**
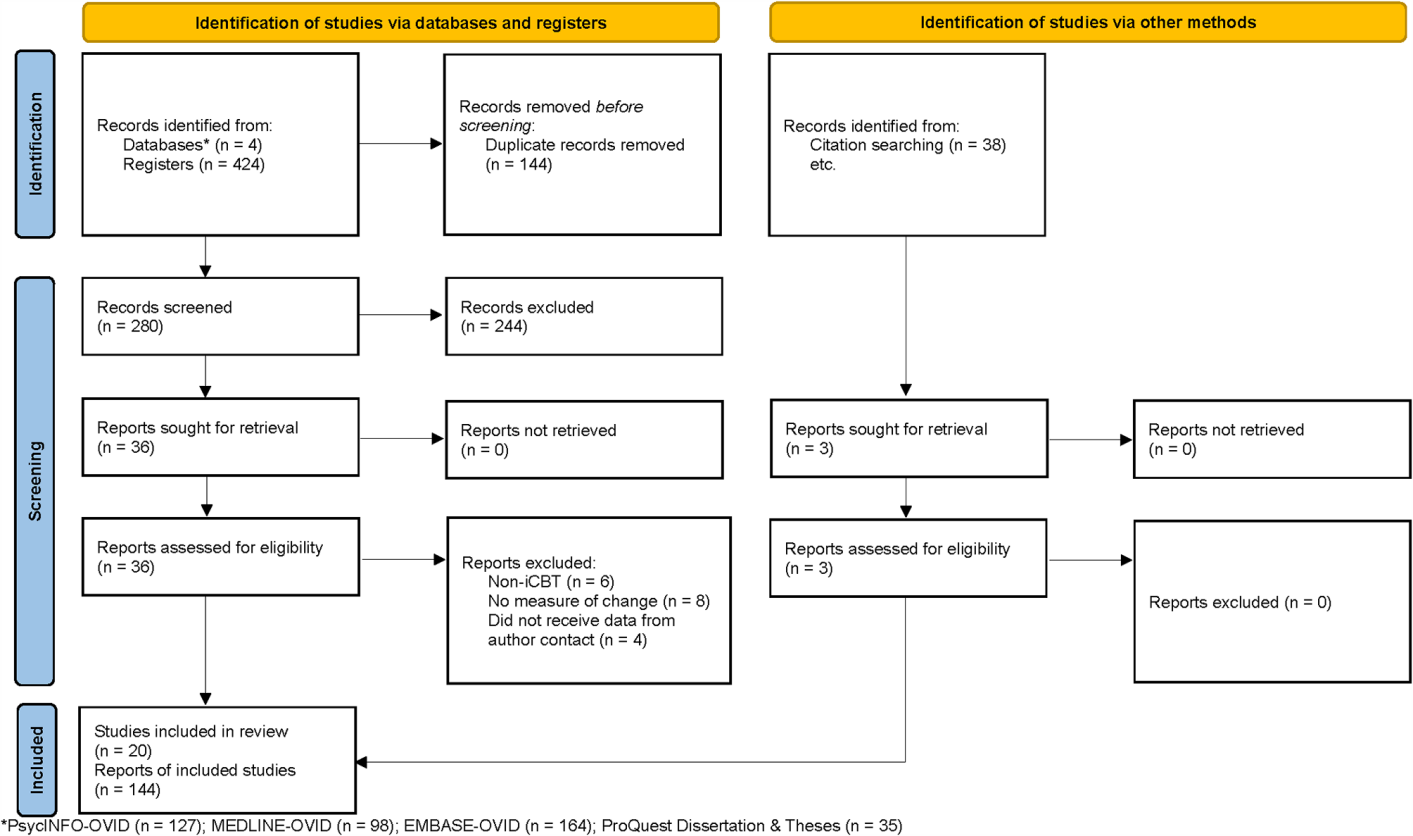
PRISMA flow diagram [18]

A total of 20 studies were included (see Fig. 1). Trained screeners (S.R. & S.S.) examined each study independently. Inter-rater reliability via percentage agreement was high at each phase (title/abstract [97.5%] and full text [95.0%]). Discrepancies were discussed in a group until consensus was reached among all authors; thus, final study selection had 100% inter-rater reliability. For each screening phase, SWIFT-Active Screener, a web-based review software, was used to screen all citations. [21].

### Data extraction

Demographics, study data, and predictor information were extracted from each study (see S1). Demographic information extracted were: (1) *age* (i.e., mean age of the study sample), and (2) *reported gender* (i.e., whether participants were primarily men, women, or mixed [multiple genders in sample]). Study data information extracted were: (1) *study condition* (iCBT or control: active [e.g., psychoeducation]; treatment as usual [TAU]; or waitlist), (2) *sample size*, (3) *pre- and post-intervention mean and standard deviations* across study conditions on all outcomes, (4) *target outcome* were distinguished by primary (e.g., PTSD targeted treatment and PTSD outcome) or secondary (e.g., PTSD targeted treatment and substance use outcome) outcomes. In the event multiple measures were taken for the same categorical outcomes (e.g., two measures of PTSD), the outcome with the largest effect size were kept. Predictor information extracted were: (1) the type of *outcome measured* (i.e., anxiety (e.g., Beck Anxiety Inventory [BAI] [22]), depression (e.g., Beck Depression Inventory [BDI] [23]), health and functioning (e.g., Brief Inventory of Psychosocial Functioning [B-IPF] [24]), PTSD (e.g., PTSD Checklist [PCL] [25]), quality of life (e.g., Quality of Life Enjoyment and Satisfaction [Q-LES-Q-SF] [26]), substance use (e.g., cigarettes smoked), and behavior (health behavior related to scales or activities; e.g., hours slept); (2) *format* (i.e., web, computer, phone, mobile app); (3) *length of intervention* (in weeks); (4) *delivery* (self-guided or facilitated); (5) *concurrent treatment allowed* (yes or no); and (6) *dropoutrate*^1^ (%).

### Data Analysis

The meta-analyses were conducted using the Comprehensive Meta-Analysis (CMA) software version 3. [27] All demographics and study data and were inserted into CMA. In addition, a correlation of 0.77 (used in a previous meta-analysis with similar design [28]) was inserted to account for between-subjects variance in within-subjects designs. For all studies, effect direction was selected to represent whether the measures of change on outcomes were in line with our hypotheses (i.e., outcomes improved over time; positive) or not (i.e., outcomes did not improve over time; negative).

Main analyses examined the combined effects of iCBT interventions and the relative effects of iCBT to control conditions across outcome measures. Hedges’ *g* effect size was used. Effect sizes were interpreted as follows, 0.41 for a minimum effect representing a practically significant effect, 1.15 for a moderate effect, and 2.70 for a strong effect. [29] Further, a continuous meta-regression was used. Following the main analyses, subsequent predictor analyses were examined across outcomes. Sub-group analyses were conducted if there are a minimum of five samples. [30] Lastly, publication bias was assessed via visual inspection of the funnel plot, Egger’s Regression test, Durval and Tweedie’s trim-and-fill, and classic fail-safe.

## Results

### Study characteristics

Our meta-analysis included 20 studies with 91 samples of data from veterans and active military members. Across samples, the gender distribution was of men only^2^ (*n* = 54) and mixed genders^3^ (*n* = 37), with no samples consisting of women only (*n* = 0). The mean age across samples was 43.66 (ranging from 28 to 67 years; see Table 1 for raw data and Table S1 for predictor information). Across samples, the distribution of samples was as follows: *n* = 56 for iCBT, and *n* = 35 for controls (active, *n* = 14; TAU, *n* = 17; and waitlist, *n* = 4). The pooled sample sizes across studies included data from 1614 individuals (iCBT [*n =* 872] and controls [active, *n* = 141; TAU, *n* = 478; and waitlist, *n* = 123]).

**Table 1:** Data of Included Studies

Study	Study Condition	Specific Control Condition	Outcome Measured	Target Outcome	*N*	*g*	*SE*	Pre-		Post	
								*M*	*SD*	*M*	*SD*
Acosta et al. (2017)	Control (TAU)	VA Care Services	Substance Use	Primary	69	0.43	0.08	27.60	24.90	17.50	20.30
	Control (TAU)	VA Care Services	PTSD	Primary	69	0.49	0.09	46.90	10.60	40.90	12.80
	Control (TAU)	VA Care Services	QoL	Secondary	69	0.40	0.08	46.90	22.50	56.20	23.00
	iCBT	--	Substance Use	Primary	55	0.12	0.09	20.10	25.20	17.30	21.90
	iCBT	--	PTSD	Primary	55	0.46	0.10	47.40	13.30	41.20	13.10
	iCBT	--	QoL	Secondary	55	0.26	0.09	52.70	22.80	59.10	25.40
Belsher et al. (2015	iCBT	--	PTSD	Primary	12	0.42	0.19	54.30	13.58	47.90	14.72
Cooper et al. (2017)	Control (Active)	Psychoeducation	Health & Functioning	Primary	34	0.63	0.12	115.68	32.94	136.32	30.65
	iCBT	--	Health & Functioning	Primary	32	0.68	0.13	105.56	38.36	130.69	27.65
Dobkin et al. (2020)	Control (TAU)	VA Care Services	Anxiety	Secondary	39	0.05	0.11	23.49	4.57	23.77	5.25
	Control (TAU)	VA Care Services	Behavior	Secondary	39	-0.16	0.11	25.31	6.98	26.69	9.04
	Control (TAU)	VA Care Services	Health & Functioning	Secondary	39	-0.14	0.11	32.01	8.98	30.65	9.40
	Control (TAU)	VA Care Services	QoL	Secondary	39	0.12	0.11	50.84	26.17	47.39	28.12
	Control (TAU)	VA Care Services	Depression	Primary	39	0.05	0.11	21.60	4.30	21.37	5.27
	iCBT	--	Depression	Primary	39	1.21	0.14	22.49	3.85	16.46	5.18
	iCBT	--	Anxiety	Secondary	39	0.76	0.12	24.15	4.14	20.10	5.55
	iCBT	--	Behavior	Secondary	39	0.80	0.12	25.16	6.02	31.39	8.15
	iCBT	--	Health & Functioning	Secondary	39	0.29	0.11	33.23	8.02	35.72	8.49
	iCBT	--	QoL	Secondary	39	0.55	0.11	47.50	20.75	59.19	20.71
Engel et al. (2015)	Control (TAU)	PTSD Care	PTSD	Primary	29	0.44	0.13	54.48	11.23	48.52	13.87
	Control (TAU)	PTSD Care	Depression	Secondary	29	0.28	0.13	11.67	4.65	10.24	5.12
	iCBT	--	PTSD	Primary	29	0.38	0.13	58.00	9.95	50.72	18.76
	iCBT	--	Depression	Secondary	29	0.39	0.13	13.53	5.43	11.00	6.65
Engel et al. (2021)	Control (Waitlist)	--	PTSD	Primary	13	0.19	0.18	33.59	15.34	30.46	15.51
	iCBT	--	PTSD	Primary	8	0.09	0.21	33.50	14.88	31.75	17.53
Herbst et al. (2018)	iCBT	--	Substance Use	Primary	20	1.19	0.19	4.40	2.20	1.80	1.90
	iCBT	--	Health & Functioning	Secondary	20	0.32	0.15	37.00	23.70	29.40	22.30
Hobfoll et al. (2016)	Control (Waitlist)	--	PTSD	Primary	94	-0.15	0.07	37.46	11.48	39.21	11.18
	Control (Waitlist)	--	Depression	Secondary	94	0.04	0.07	12.40	5.36	12.17	5.08
	iCBT	--	PTSD	Primary	209	0.37	0.05	40.03	11.19	35.95	10.93
	iCBT	--	Depression	Secondary	209	0.25	0.05	12.45	5.36	11.11	5.52
Litz et al. (2007)	Control (Active)	Supportive Counselling	Anxiety	Secondary	17	0.55	0.17	20.92	15.00	12.59	13.45
	Control (Active)	Supportive Counselling	Depression	Secondary	17	0.57	0.17	24.43	12.08	17.47	11.19
	Control (Active)	Supportive Counselling	PTSD	Primary	17	0.80	0.18	29.16	9.93	20.00	11.50
	iCBT	--	Anxiety	Secondary	14	0.92	0.21	18.70	10.60	8.43	5.93
	iCBT	--	Depression	Secondary	14	0.66	0.19	18.87	9.52	12.14	9.56
	iCBT	--	PTSD	Primary	14	0.88	0.20	26.71	9.02	14.86	13.35
Mackintosh et al. (2017)	Control (Active)	Anger Management Treatment	PTSD	Primary	30	2.39	0.24	50.30	19.70	43.40	12.30
	Control (Active)	Anger Management Treatment	Depression	Secondary	30	0.46	0.13	14.00	7.34	8.30	12.30
	Control (Active)	Anger Management Treatment	Health & Functioning	Secondary	30	0.15	0.12	3.30	1.69	2.60	4.27
	Control (Active)	Anger Management Treatment	Anxiety	Primary	30	0.74	0.14	48.90	8.05	38.30	14.20
	Control (Active)	Anger Management Treatment	Health & Functioning	Secondary	30	0.50	0.13	17.20	4.11	12.60	8.65
	iCBT	--	Anxiety	Primary	28	0.81	0.14	46.40	9.05	37.90	10.80
	iCBT	--	Health & Functioning	Secondary	28	0.40	0.13	15.50	3.70	10.70	10.30
	iCBT	--	PTSD	Primary	28	-0.19	0.13	43.30	17.90	32.30	49.40
	iCBT	--	Depression	Secondary	28	0.13	0.13	12.30	5.40	8.90	21.50
	iCBT	--	Health & Functioning	Secondary	28	-0.07	0.12	2.80	1.80	2.30	5.70
Mohr et al. (2011)	Control (TAU)	Outpatient Clinic	Depression	Primary	29	0.38	0.13	19.23	3.72	17.27	5.29
	iCBT	--	Depression	Primary	20	0.64	0.16	20.83	3.96	16.71	6.42
Nelson et al. (2014)	iCBT	--	Depression	Primary	19	0.28	0.15	21.86	11.11	18.21	13.20
	iCBT	--	Anxiety	Secondary	19	0.39	0.16	16.36	12.42	11.57	10.15
	iCBT	--	Health & Functioning	Secondary	19	0.33	0.15	25.93	11.68	29.86	10.75
Pfeiffer et al. (2020)	Control (TAU)	Primary or Integrated Care	Depression	Primary	128	0.43	0.06	13.40	3.60	11.70	4.10
	Control (TAU)	Primary or Integrated Care	Health & Functioning	Secondary	128	0.40	0.06	32.10	9.90	36.20	10.30
	Control (TAU)	Primary or Integrated Care	QoL	Secondary	128	0.18	0.06	38.80	8.70	40.50	9.90
	iCBT	--	Depression	Primary	108	0.65	0.07	14.00	3.90	11.10	4.70
	iCBT	--	Health & Functioning	Secondary	108	0.54	0.07	31.40	9.70	37.30	11.50
	iCBT	--	QoL	Secondary	108	0.41	0.07	37.70	8.30	41.60	9.90
Possemato et al. (2016)	Control (Active)	Self-Managed PTSD Coach	PTSD	Primary	10	0.33	0.20	56.00	15.30	49.80	18.10
	Control (Active)	Self-Managed PTSD Coach	Depression	Secondary	10	0.26	0.20	11.30	9.70	8.70	8.30
	Control (Active)	Self-Managed PTSD Coach	QoL	Secondary	10	-0.35	0.20	63.30	29.20	52.60	25.50
	iCBT	--	PTSD	Primary	10	0.98	0.25	51.00	7.70	40.00	10.90
	iCBT	--	Depression	Secondary	10	0.32	0.20	11.60	6.70	9.40	5.50
	iCBT	--	QoL	Secondary	10	0.50	0.21	37.50	19.70	47.70	16.10
Possemato et al. (2019)a	iCBT	--	PTSD	Primary	9	0.41	0.21	48.20	11.61	41.78	14.90
	iCBT	--	Substance Use	Secondary	9	0.16	0.21	12.60	8.10	11.17	7.58
	iCBT	--	QoL	Secondary	9	0.54	0.22	47.76	19.39	63.45	27.62
	iCBT	--	Health & Functioning	Secondary	9	0.79	0.24	1.08	0.40	1.63	0.65
	iCBT	--	Depression	Primary	9	0.17	0.21	59.31	13.03	62.23	15.99
Possemato et al. (2019)b	iCBT	--	PTSD	Primary	11	0.63	0.21	51.87	9.01	43.16	13.42
	iCBT	--	Substance Use	Secondary	11	0.27	0.19	13.30	8.70	10.77	8.52
	iCBT	--	QoL	Secondary	11	0.47	0.20	43.78	16.42	53.42	20.10
	iCBT	--	Health & Functioning	Secondary	11	0.47	0.20	1.44	0.49	1.76	0.67
	iCBT	--	Depression	Primary	11	0.24	0.19	57.47	12.45	60.99	14.34
Pulantara et al. (2018)	iCBT	--	Behavior	Primary	27	2.13	0.23	15.59	4.13	5.63	4.76
	iCBT	--	PTSD	Secondary	27	0.81	0.15	38.41	14.10	27.22	11.87
	iCBT	--	Depression	Secondary	27	0.88	0.15	8.41	5.22	3.63	5.34
	iCBT	--	Anxiety	Secondary	27	0.60	0.14	6.17	5.32	2.91	2.94
Stecker et al. (2014)	Control (TAU)	Access to Usual Services	PTSD	Primary	151	0.37	0.06	59.70	11.70	55.00	13.10
	Control (TAU)	Access to Usual Services	Depression	Secondary	151	0.54	0.06	16.40	4.80	13.80	4.80
	iCBT	--	PTSD	Primary	123	0.61	0.07	59.20	11.80	51.30	13.60
	iCBT	--	Depression	Secondary	123	0.66	0.07	16.30	4.70	12.80	5.60
Taylor et al. (2017)	Control (Active)	In-Person CBT	Behavior	Primary	34	2.95	0.27	72.50	2.10	79.40	2.40
	Control (TAU)	Phone Call Assessments	Behavior	Primary	33	0.09	0.12	72.90	2.20	73.10	2.30
	iCBT	--	Behavior	Primary	33	5.02	0.43	73.20	2.20	84.50	2.20
Timmons (1997)	Control (Active)	Stress Inoculation Training	Anxiety	Primary	16	0.65	0.18	62.25	10.09	55.56	6.40
	Control (Waitlist)	--	Anxiety	Primary	16	-0.20	0.16	60.75	9.84	63.00	11.54
	iCBT	--	Anxiety	Primary	16	0.40	0.17	60.25	10.90	55.94	8.42
Voorhees et al. (2012)	iCBT	--	Depression	Secondary	50	0.37	0.10	8.90	4.00	7.30	4.40
	iCBT	--	PTSD	Primary	50	0.27	0.10	35.00	10.80	32.00	11.40
	iCBT	--	QoL	Secondary	50	0.01	0.09	44.60	10.80	44.50	11.40

*Notes.* iCBT = internet based cognitive behavioral therapy; PTSD = posttraumatic stress disorder; QoL = quality of life

### Overall analyses

First, we examined the effects of iCBT and control interventions on all outcomes. A total of 91 samples were entered into the mixed, random effects model to determine the pooled effect of iCBT interventions relative to control groups on primary and secondary outcomes. Results indicated a significant difference across groups, *Q*(3) = 50.30, *p* < .001. The pooled effect size showed a small but meaningful effect for iCBT, *g* = 0.54, *SE* = 0.04, 95% *CI* (0.45, 0.62), *Z* = 12.32, *p* < .001. These effects were robust relative to the effects of controls - TAU, *g* = 0.26, *SE* = 0.05, 95% *CI* (0.17, 0.36), *Z* = 5.25, *p* < .001, and waitlist, *g* = -0.04, *SE* = 0.08, 95% *CI* (-0.19, 0.11), *Z* = -0.56, *p* = .58. However, for studies with an active alternative intervention, the pooled effects were observed to be slightly larger in magnitude^4^, relative to the iCBT, *g* = 0.59, *SE* = 0.13, 95% *CI* (0.34, 0.84), *Z* = 4.61, *p* < .001. Across all samples, effects were found to be considerably heterogeneous (*I*^2^ = 87.96), *Q*(90) = 747.62, *p* < .001.

### Effects of iCBT on primary outcomes across outcomes measured

**Fig. 2:**
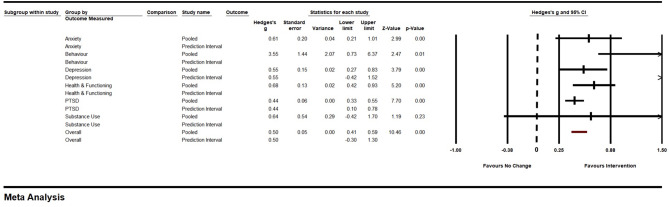
Forest Plot of Primary Outcome across Outcomes Measured for iCBT Group

Next, we examined the effects of iCBT interventions on the primary outcomes of each study. Across all primary outcomes measured, a total of 25 samples were entered into the mixed, random effects model to determine the pooled effect of iCBT intervention. The effect size for primary outcomes showed a small but meaningful effect for iCBT, *g* = 0.50, *SE* = 0.05, 95% *CI* (0.41, 0.59), *Z* = 10.46, *p* < .001 (see Fig. 2). Subgroup analyses (between groups with adequate sample representation) on examining the primary outcomes by the types of outcomes measured (PTSD and depression) found no significant differences across groups, *Q*(1) = 0.49, *p* = .48. Specifically, the effects were relatively similar between depression [*g* = 0.55, *SE* = 0.15, 95% *CI* (0.27, 0.84), *Z* = 3.79, *p* < .001] and PTSD [*g* = 0.44, *SE* = 0.06, 95% *CI* (0.33, 0.55), *Z* = 7.70, *p* < .001]. The remaining outcomes (anxiety, behavior, health and functioning, quality of life, and substance use) were not examined due to low sample size.

### Effects of iCBT on secondary outcomes across outcomes measured

**Fig. 3:**
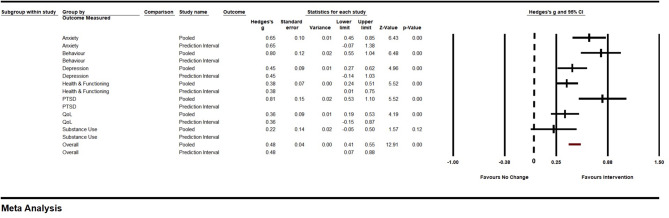
Forest Plot of Secondary Outcome across Outcomes Measured for iCBT Group

Next, we examined the effects of iCBT intervention on the secondary outcomes of each study. Across all outcomes measured, a total of 31 samples were entered into the mixed, random effects model to determine the pooled effect of iCBT intervention. The effect size for secondary outcomes showed a small but meaningful effect for iCBT, *g* = 0.48, *SE* = 0.04, 95% *CI* (0.41, 0.55), *Z* = 12.91, *p* < .001 (see Fig. 3). Subgroup analyses (between groups with adequate sample representation) on examining the secondary outcomes by outcome measured (depression, health and functioning, and quality of life) found no significant differences across groups, *Q*(2) = 0.56, *p* = .76. Specifically, depression showed a small but meaningful effect, *g* = 0.45, *SE* = 0.09, 95% *CI* (0.27, 0.62), *Z* = 4.96, *p* < .001. However, effects were non-meaningful for health and functioning (*g* = 0.38, *SE* = 0.07, 95% *CI* (0.25, 0.51), *Z* = 5.52, *p* < .001), and quality of life (*g* = 0.36, *SE* = 0.09, 95% *CI* (0.19, 0.53), *Z* = 4.19, *p* < .001). The remaining outcomes (anxiety, behavior, PTSD, and substance use) were not examined due to low sample size.

### Meta-regression

A continuous meta-regression was conducted using the moments method as the estimation framework to determine their influence on the pooled effects within iCBT samples [27]. Continuous predictors included age, dropout rate and length of intervention. This model did not account for significant variance within the data. Within Model 1, only length of intervention significantly predicted the study variance, with shorter lengths of interventions having a larger magnitude of effects (see Table 2).

**Table 2:** Meta-Regression Test Statistics

Model	*B*	*SE*	95% *CI*		*Z*	*Q*	*df*
			Lower	Upper			
**Continuous Predictors**	--	--	--	--	--	8.64*	3
Age	0.01	0.00	-0.00	0.01	1.33	--	--
Dropout Rate	-0.00	0.00	-0.01	0.01	-0.32	--	--
Length of Intervention	-0.04	0.01	-0.07	-0.02	-2.87**	--	--

*Notes*. *B* = coefficient; *SE* = standard error; *CI* = confidence interval; *Z* = Fisher’s Z; *Q* = *Q-*statistics (Cochran’s observed dispersion); *df* = degree of freedom; *p* = *p*-value

* *p* < .05, ** *p < .01.*

### Effects of iCBT on Study Outcomes across categorical predictors

#### Overall

We examined whether the effects of iCBT (*k* = 56) varied as a result of categorical predictors (concurrent treatment, delivery, format, gender, outcomes measured) via *Q*-statistics. [30] Analyses with all outcomes in iCBT group found that concurrent treatment resulted in different effect sizes across sub-groups, while delivery, format, gender, and outcomes measured did not result in different effects across outcomes. Specifically, studies that extended participant eligibility to allow concurrent treatments yielded a small positive effect, while studies that did not allow concurrent treatment did not meet the effect threshold (see Table 3).

**Table 3:** Meta-Analysis Test Statistics on Sub-Group Analyses Across Predictors for All Outcomes in iCBT Group (k = 56)

Intervention Outcome	*k*	*g*	*SE*	95% *CI*		*Z*	*Q*
				Lower	Upper		
**Concurrent Treatment**							7.84**
Yes	37	0.62^a^	0.06	0.50	0.74	10.04***	
No	19	0.40	0.05	0.30	0.50	7.89***	
**Delivery**							0.42
Facilitated	33	0.52^a^	0.04	0.44	0.61	12.11***	
Self-Guided	23	0.58^a^	0.08	0.42	0.74	7.23***	
**Format**							5.33
Web	26	0.46^a^	0.07	0.33	0.58	6.99***	
Mobile App	14	0.64^a^	0.12	0.41	0.88	5.31***	
Computer	13	0.56^a^	0.06	0.44	0.68	9.16***	
Phone^	3	--	--	--	--	--	
**Gender**							3.21
Males	33	0.47^a^	0.05	0.37	0.57	8.93***	
Mixed	23	0.63^a^	0.07	0.49	0.77	8.86***	
Females^	0	--	--	--	--	--	
**Outcomes Measured**							11.64
Depression	14	0.49^a^	0.08	0.34	0.65	6.33***	
PTSD	13	0.47^a^	0.06	0.36	0.59	8.01***	
Health & Functioning	9	0.42^a^	0.07	0.28	0.55	6.10***	
Quality of Life	7	0.36	0.09	0.19	0.53	4.19***	
Anxiety	6	0.64^a^	0.08	0.48	0.80	7.77***	
Substance Use^	4	--	--	--	--	--	
Behavior^	3	--	--	--	--	--	

*Notes*. *k* = number of samples; *g* = Hedges’ *g*; *SE* = standard error; *CI* = confidence interval; *Z* = Fisher’s *Z*; *Q* = *Q-*statistics (Cochran’s observed dispersion); ^ = predictor with less than 5 samples were not examined

* *p* < .05; ** *p* < .01; *** *p* < .001

^a^ = minimum effect representing a practically significant effect

#### Primary outcomes

We examined whether the effects of iCBT on primary outcomes (*k =* 25) varied across categorical predictors (concurrent treatment, delivery, format, and gender) via *Q*-statistics. Analyses with primary outcomes in iCBT groups found that concurrent treatment and gender resulted in different effect sizes across sub-groups, while delivery and format did not result in different effects across outcomes. Specifically, studies that extended participant eligibility to allow concurrent treatments yielded a marginally stronger effect relative to studies that did not allow concurrent treatment. For gender, samples containing mixed-gender populations yielded more robust effects relative to samples that only contained males (see Table 4).

**Table 4:** Meta-Analysis Test Statistics on Sub-Group Analyses Across Predictors for Primary Outcomes in iCBT Group (k = 25)

Intervention Outcome & Moderators	*k*	*g*	*SE*	95% *CI*		*Z*	*Q*
				Lower	Upper		
**Concurrent Treatment**							6.12*
Yes	15	0.88^a^	0.14	0.61	1.15	6.39***	
No	10	0.48^a^	0.09	0.30	0.65	5.35***	
**Delivery**							2.30
Facilitated	14	0.61^a^	0.08	0.46	0.76	7.91***	
Self-Guided	11	0.89^a^	0.17	0.56	1.21	5.29***	
**Format**							2.24
Web	13	0.59^a^	0.13	0.35	0.84	4.69***	
Mobile App	5	1.09^a^	0.32	0.46	1.72	3.38**	
Computer	5	0.67^a^	0.14	0.39	0.94	4.79***	
Phone^	2	--	--	--	--	--	
**Gender**							4.61*
Males	16	0.56^a^	0.10	0.38	0.75	5.93***	
Mixed	9	0.97^a^	0.17	0.65	1.30	5.90***	
Females^	0	--	--	--	--	--	

*Notes*. *k* = number of samples; *g* = Hedges’ *g*; *SE* = standard error; *CI* = confidence interval; *Z* = Fisher’s *Z*; *Q* = *Q-*statistics (Cochran’s observed dispersion); ^ = predictor with less than 5 samples were not examined

* *p* < .05; ** *p* < .01; *** *p* < .001

^a^ = minimum effect representing a practically significant effect (Ferguson, 2009)

#### Secondary outcomes

We examined whether the effects of iCBT on secondary outcomes (*k =* 31) varied across categorical predictors (concurrent treatment, delivery, format, and gender) via *Q*-statistics. Analyses with secondary outcomes in iCBT groups found that concurrent treatment and format resulted in different effect sizes across sub-groups, while delivery and gender did not result in different effects across outcomes. Specifically, studies that extended participant eligibility to allow concurrent treatments met the threshold for practically significant effects. For format, studies that used computers yielded higher effects than those that used mobile applications (see Table 5).

**Table 5:** Meta-Analysis Test Statistics on Sub-Group Analyses Across Predictors for Secondary Outcomes in iCBT Group (k = 31)

Intervention Outcome & Moderators	*k*	*g*	*SE*	95% *CI*		*Z*	*Q*
				Lower	Upper		
**Concurrent Treatment**							4.25*
Yes	22	0.52^a^	0.06	0.40	0.64	8.57***	
No	9	0.35	0.06	0.24	0.46	6.21***	
**Delivery**							0.76
Facilitated	19	0.50^a^	0.05	0.39	0.60	9.44***	
Self-Guided	12	0.42^a^	0.07	0.28	0.56	5.77***	
**Format**							8.34*
Web	13	0.39	0.07	0.26	0.52	5.89***	
Mobile App	9	0.46^a^	0.10	0.26	0.66	4.47***	
Computer	8	0.52^a^	0.06	0.40	0.64	8.56***	
Phone^	1	--	--	--	--	--	
**Gender**							0.67
Males	17	0.43^a^	0.06	0.31	0.55	6.97***	
Mixed	14	0.50^a^	0.07	0.37	0.63	7.52***	
Females^	0	--	--	--	--	--	

*Notes*. *k* = number of samples; *g* = Hedges’ *g*; *SE* = standard error; *CI* = confidence interval; *Z* = Fisher’s *Z*; *Q* = *Q-*statistics (Cochran’s observed dispersion); ^ = predictor with less than 5 samples were not examined

* *p* < .05; ** *p* < .01; *** *p* < .001

^a^ = minimum effect representing a practically significant effect (Ferguson, 2009)

### Publication Bias

**Fig. 4:**
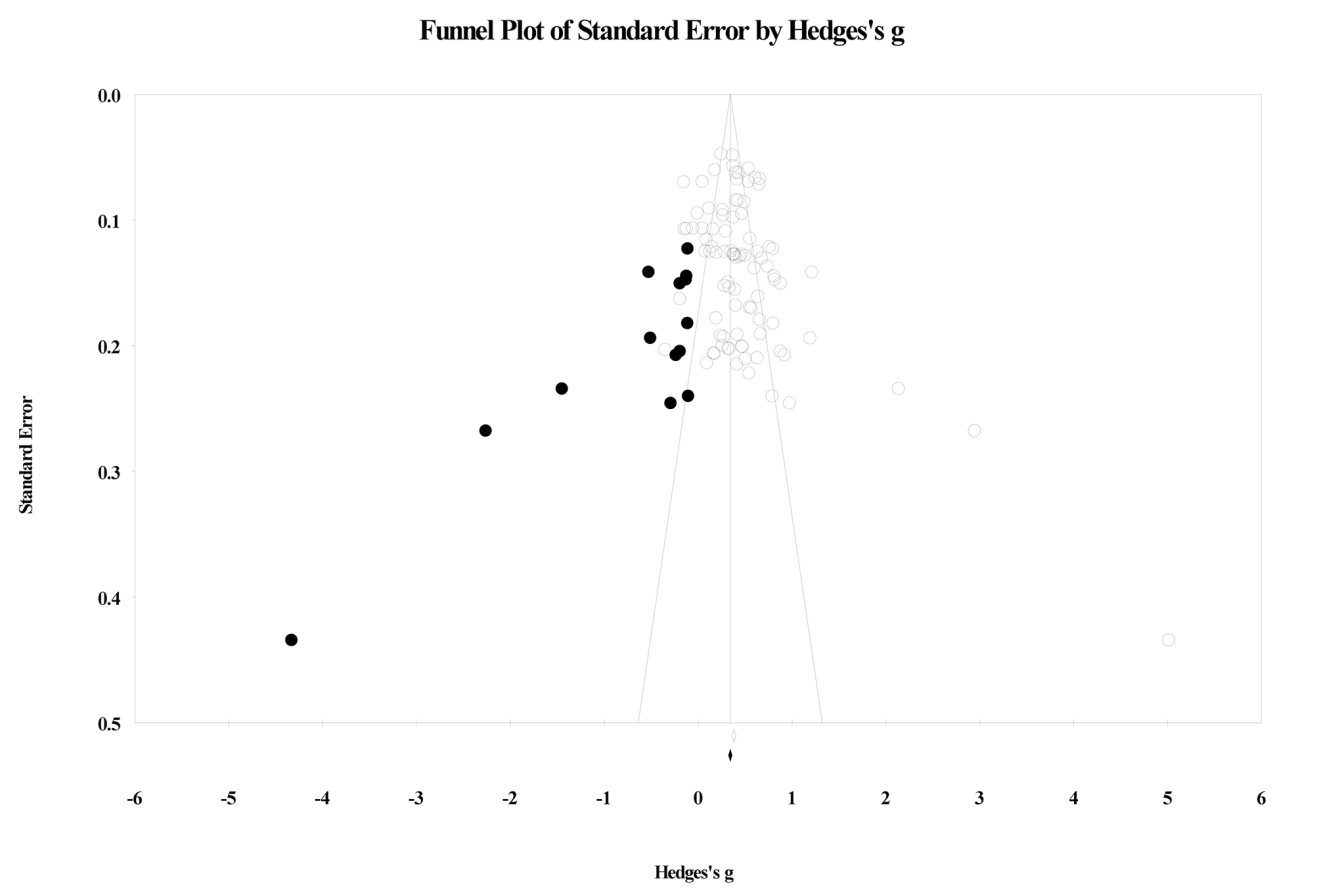
Funnel Plot across all included studies

Visual inspection of the funnel plot indicates that a number of studies are clustered towards the center, with a small number scattered to the right and some imputed studies to the left, suggesting publication bias (see Fig. 4). Egger’s regression test detected a significant asymmetry, (*B*_0_) = 1.95, *t*(89) = 2.84, *p* = .005. For Duval and Tweedie’s (2000) trim-and-fill, under the random-effects model, the point estimate and 95% confidence interval for the combined studies is 0.46 (0.39, 0.52). Using trim-and-fill, the imputed point estimate is 0.34 (0.25, 0.41). Classic fail-safe N revealed that it would take 27,431 ‘null’ studies in order for the combined 2-tailed *p*-value to exceed 0.05. In other words, there would need to be 301.4 missing studies for every observed study for the effect to be nullified. Taken together, analyses examining publication bias suggest the presence of some biases in that study effects may be over-represented and skewed towards positive, but that these biases, if any, should be marginal in nature.

## Discussion

The current meta-analysis sought to determine the relative effectiveness of iCBT for military and veteran populations. Results found iCBT to be more effective across a range of outcome measures when compared against waitlist controls and TAU conditions, but not when compared to active alternative interventions. The pooled effect sizes of iCBT interventions were small, but of practical significance and meaningful importance. [29] Internet-based CBT were effective for a number of mental health conditions, with results robust for both primary (PTSD and depression) and secondary (depression) outcomes.

### Effectiveness of iCBT for military populations

The effects of iCBT treatments were robust across a number of primary and secondary study outcomes, and comparable to prior meta-analyses of iCBT treatments conducted with non-military samples on depression (*d* = 0.41) [11] and PTSD (*d* = -0.60) [42]. This underscores the robustness of iCBT treatments. In addition, our results (*g* = 0.54) were similar in magnitude to the previous findings on CBT treatments for depression (*g* = 0.71) [43] and iCBT for PTSD (*g* = 0.72) [8] among the general population. Although slightly smaller in size by comparison, the relative comparability of these findings establishes the validity of CBT treatments generally, and its adaptability to diverse modes of delivery, including internet, app, and telephone-based deliveries.

Yet, the effects of iCBT were not superior when compared against groups receiving alternative interventions. This, in part, may be due to variability in treatments considered “active alternative controls”. In our sample of studies, these groups ranged from psychoeducation[31]–[32], supportive counseling [33], in-person therapy [18], and group counseling [34]. The size of the effects within this category are not uniform or homogenous. Instead, the effects are more indicative of diverse treatments received by participants. Indeed, prior reviews note similar considerations, underscoring the superiority of CBT to be highly dependent on the nature of the control conditions tested against. [35].

### Considerations for the use of iCBT with military populations

In addition to the pooled effects, iCBT interventions were found to be differentially effective based on several factors. For participants receiving other treatments concurrently with iCBT, such as medications and adjunctive therapies, improvements in symptoms were of greater magnitude relative to those not receiving concurrent treatments. Although this may not be surprising given the cumulative effects of treatments, it also suggests that iCBT may be a complementary treatment when used with others. This has important implications for military and veteran communities in particular, due to the higher prevalence of physical and mental health comorbidities. [36] When engaging in treatment planning, the option to add a course of iCBT, if appropriate, may have advantageous effects. Future research could explore the additive effects of iCBT, whether as the primary or supplementary form of therapy.

While the format in which iCBT was presented (i.e., computer versus mobile and web-based applications) leads to differences in overall effects, the overall effect sizes of were comparable. Meanwhile, in accordance with prior research observing differences between facilitated versus self-guided iCBT programs [37], the current meta-analysis also found significant differences with small effects, though these effects were similar in size. However, these differences may be driven largely by the inclusion of CBT for insomnia, which are exclusively self-guided and have the largest effect sizes.

Results found that iCBT interventions that included both men and women participants were found to have larger effects than male only participants. Historically, diverse genders have been excluded from service, and underrepresented in military research. [38] The increase in effects from mixed gender studies may be reflective of more recent research, and may further be confounded with advancements in technology and improvements in program design, both of which may result in a more efficacious treatment program. Despite these speculations, prior research has underscored gender differences in military and veteran samples following mental health treatments. [39, 40] Finally, shorter iCBT interventions were generally found to have greater magnitude of effects. [44] This may be due to shorter programs being more manageable with less participant attrition. This may also explain the lack of relationship observed between attrition rate and study effects. Thus, future studies should examine these factors in relation to iCBT intervention effectiveness.

Taken together, these considerations provide the deeper contexts for understanding the effectiveness of iCBT for military and veteran populations. While findings are positive, more research is needed to better evaluate the relative weights of various population and intervention characteristics, such as examinations of fidelity [41], treatment adherence, and individual motivations. Importantly, these factors point to considerations and areas of negotiation when engaging in treatment planning and offer insights into the interpretations of treatment efficacy. Finally, results from the current meta-analysis should be considered in lieu of the presence of potential publication biases that may skew the effects of iCBT. As technologies continue to evolve and demands for telehealth and remote delivery of services amplify, we expect that both the quality of evidence and sophistication of treatment will increase over time. As such, continued research is needed to better position the use of iCBT for military personnel and veterans and other high-risk populations with unique service needs.

### Conclusions and future directions

With the changing landscape of health service delivery brought on by the pandemic, investigations into telehealth delivery of services may be more relevant than ever before. The current meta-analysis is one of few to investigate the utility of iCBT for military personnel and veterans. Together, findings underscore the potential utility and effectiveness of iCBT interventions for military and veteran populations. Specifically, iCBT may be optimized under conditions of inclusive enrollments (e.g., mixed genders, allowance for concurrent treatments). Clinicians may feel comfortable recommending access to iCBT treatments for military and veteran populations, especially as an adjunctive, add-on, or while awaiting in-person treatment(s). Meanwhile, future research should explore the benefits and obstacles of iCBT through rigorous investigations of the barriers and facilitators of successful deliveries of services. Further, while results from this meta-analysis was not pre-registered (e.g., PROSPERO), future reviews may wish to consider this approach.

## Electronic supplementary material

Below is the link to the electronic supplementary material.

Supplementary Materials for A meta-analysis of internet-based cognitive behavioral therapy for military and veteran populations

## Data Availability

The datasets generated and/or analysed during the current study are available in Supplementary files.

## Footnotes

1 Dropout rate is noted as the number of participants that did not complete the treatment (where information was provided).

2 Study samples with > 92% males were categorized as males only for the purpose of analyses.

3 Study samples containing > 8% males were categorized as mixed genders for the purpose of analyses.

4 Effects of active control were also larger (g = 0.57) in comparison to iCBT after removing two studies where the active control were in-person deliveries of the same protocol.

## List of abbreviations

BAI: Beck Anxiety Inventory
BDI: Beck Depression Inventory
B-IPF: Brief Inventory of Psychosocial Functioning
CMA: Comprehensive Meta-Analysis
iCBT: internet-based cognitive behavioral therapy
PTSD: posttraumatic stress disorder
PRISMA: Preferred Reporting Items for Systematic Reviews and Meta-Analyses
PCL: posttraumatic stress disorder checklist
Q-LES-Q-SF: Quality of Life Enjoyment and Satisfaction
TAU: treatment-as-usual
