# Supplementary material for: A meta-analysis of internet-based cognitive behavioral therapy for military and veteran populations
Source: BMC Psychiatry. 2023 Apr 3;23:223. doi: 10.1186/s12888-023-04668-1 (PMC10068715; doi:10.1186/s12888-023-04668-1)
Supplement: Supplementary file 1 — Supplementary Materials for A meta-analysis of internet-based cognitive behavioral therapy for military and veteran populations [file 12888_2023_4668_MOESM1_ESM.docx]

**Supplementary Material**

**Search Term Strings**

1. (internet or web or online or computer or computerized or apps or application or digital).mp. [mp=title, abstract, original title, name of substance word, subject heading word, floating sub-heading word, keyword heading word, organism supplementary concept word, protocol supplementary concept word, rare disease supplementary concept word, unique identifier, synonyms]
2. (CBT or cognitive behavioral therapy or cognitive therapy or behavioral therapy).mp. [mp=title, abstract, original title, name of substance word, subject heading word, floating sub-heading word, keyword heading word, organism supplementary concept word, protocol supplementary concept word, rare disease supplementary concept word, unique identifier, synonyms]
3. (icbt or internet based cognitive behavioral therapy).mp. [mp=title, abstract, original title, name of substance word, subject heading word, floating sub-heading word, keyword heading word, organism supplementary concept word, protocol supplementary concept word, rare disease supplementary concept word, unique identifier, synonyms]
4. 2 or 3
5. (veteran or military or combat).mp. [mp=title, abstract, heading word, table of contents, key concepts, original title, tests & measures, mesh]
6. 1 and 4 and 5

Table S1. *Moderator Information for Included Studies (iCBT groups only)*

| **Study** | **Age** | **Gender** | **Outcome Measured** | **Primary Condition Targeted** | **Format** | **Length** | **Delivery** | **Concurrent Treatment**  **Allowed** | **Dropout Rate** |
| --- | --- | --- | --- | --- | --- | --- | --- | --- | --- |
| Acosta et al  (2017) | 34 | Males | Substance Use | Substance Use | Web | 12 | Self-Guided | No | 9 |
|  | 34 | Males | Substance Use | Substance Use | Web | 12 | Self-Guided | No | 9 |
|  | 34 | Males | Substance Use | Substance Use | Web | 12 | Self-Guided | No | 9 |
|  | 34 | Males | PTSD | Substance Use | Web | 12 | Self-Guided | No | 9 |
|  | 34 | Males | QoL | Substance Use | Web | 12 | Self-Guided | No | 9 |
|  | 34 | Males | QoL | Substance Use | Web | 12 | Self-Guided | No | 9 |
|  | 34 | Males | QoL | Substance Use | Web | 12 | Self-Guided | No | 9 |
|  | 34 | Males | QoL | Substance Use | Web | 12 | Self-Guided | No | 9 |
|  | 34 | Males | Substance Use | Substance Use | -- | -- | -- | -- | -- |
|  | 34 | Males | Substance Use | Substance Use | -- | -- | -- | -- | -- |
|  | 34 | Males | Substance Use | Substance Use | -- | -- | -- | -- | -- |
|  | 34 | Males | PTSD | Substance Use | -- | -- | -- | -- | -- |
|  | 34 | Males | QoL | Substance Use | -- | -- | -- | -- | -- |
|  | 34 | Males | QoL | Substance Use | -- | -- | -- | -- | -- |
|  | 34 | Males | QoL | Substance Use | -- | -- | -- | -- | -- |
|  | 34 | Males | QoL | Substance Use | -- | -- | -- | -- | -- |
| Belsher et al  (2015) | 35 | Males | PTSD | PTSD | Web | 8 | Facilitated | No | 33 |
| Cooper et al  (2017) | 32 | Males | Health & Functioning | mTBI | Computer | 6 | Facilitated | Yes | 21.9 |
|  | 32 | Males | Behavior | mTBI | -- | -- | -- | -- | -- |
| Dobkin et al  (2020) | 67 | Males | Depression | Depression | Web | 10 | Facilitated | Yes | 13.33 |
|  | 67 | Males | Depression | Depression | Web | 10 | Facilitated | Yes | 13.33 |
|  | 67 | Males | Anxiety | Depression | Web | 10 | Facilitated | Yes | 13.33 |
|  | 67 | Males | Depression | Depression | Web | 10 | Facilitated | Yes | 13.33 |
|  | 67 | Males | Behavior | Depression | Web | 10 | Facilitated | Yes | 13.33 |
|  | 67 | Males | Health & Functioning | Depression | Web | 10 | Facilitated | Yes | 13.33 |
|  | 67 | Males | QoL | Depression | Web | 10 | Facilitated | Yes | 13.33 |
|  | 67 | Males | Depression | Depression | -- | -- | -- | -- | -- |
|  | 67 | Males | Depression | Depression | -- | -- | -- | -- | -- |
|  | 67 | Males | Anxiety | Depression | -- | -- | -- | -- | -- |
|  | 67 | Males | Depression | Depression | -- | -- | -- | -- | -- |
|  | 67 | Males | Behavior | Depression | -- | -- | -- | -- | -- |
|  | 67 | Males | Health & Functioning | Depression | -- | -- | -- | -- | -- |
|  | 67 | Males | QoL | Depression | -- | -- | -- | -- | -- |
| Engel et al  (2015) | 36 | Mixed | PTSD | PTSD | Web | 6 | Self-Guided | No | 19 |
|  | 36 | Mixed | Depression | PTSD | Web | 6 | Self-Guided | No | 19 |
|  | 36 | Mixed | Depression | PTSD | Web | 6 | Self-Guided | No | 19 |
|  | 36 | Mixed | PTSD | PTSD | -- | -- | -- | -- | -- |
|  | 36 | Mixed | Depression | PTSD | -- | -- | -- | -- | -- |
|  | 36 | Mixed | Depression | PTSD | -- | -- | -- | -- | -- |
| Engel et al  (2021) | 38 | Males | PTSD | PTSD | Web | 5 | Facilitated | No | 60 |
|  | 38 | Males | PTSD | PTSD | -- | -- | -- | -- | -- |
| Herbst et al  (2018) | 41 | Males | Substance Use | Substance Use | Mobile App | 8 | Self-Guided | Yes | 35 |
|  | 41 | Males | Substance Use | Substance Use | Mobile App | 8 | Self-Guided | Yes | 35 |
|  | 41 | Males | Substance Use | Substance Use | Mobile App | 8 | Self-Guided | Yes | 35 |
|  | 41 | Males | Health & Functioning | Substance Use | Mobile App | 8 | Self-Guided | Yes | 35 |
| Hobfoll et al  (2016) | 34 | Mixed | PTSD | PTSD | Web | 6 | Self-Guided | Yes | 24 |
|  | 34 | Mixed | Depression | PTSD | Web | 6 | Self-Guided | Yes | 24 |
|  | 34 | Mixed | PTSD | PTSD | -- | -- | -- | -- | -- |
|  | 34 | Mixed | Depression | PTSD | -- | -- | -- | -- | -- |
| Litz et al  (2007) | 40 | Mixed | Anxiety | PTSD | Web | 8 | Facilitated | Yes | 30 |
|  | 40 | Mixed | Depression | PTSD | Web | 8 | Facilitated | Yes | 30 |
|  | 40 | Mixed | PTSD | PTSD | Web | 8 | Facilitated | Yes | 30 |
|  | 40 | Mixed | Anxiety | PTSD | -- | -- | -- | -- | -- |
|  | 40 | Mixed | Depression | PTSD | -- | -- | -- | -- | -- |
|  | 40 | Mixed | PTSD | PTSD | -- | -- | -- | -- | -- |
| Mackintosh et al  (2017) | 53 | Males | Anxiety | Anger | Mobile App | 12 | Facilitated | No | 11 |
|  | 53 | Males | Anxiety | Anger | Mobile App | 12 | Facilitated | No | 11 |
|  | 53 | Males | Health & Functioning | Anger | Mobile App | 12 | Facilitated | No | 11 |
|  | 53 | Males | PTSD | Anger | Mobile App | 12 | Facilitated | No | 11 |
|  | 53 | Males | Depression | Anger | Mobile App | 12 | Facilitated | No | 11 |
|  | 53 | Males | Health & Functioning | Anger | Mobile App | 12 | Facilitated | No | 11 |
|  | 53 | Males | Anxiety | Anger | -- | -- | -- | -- | -- |
|  | 53 | Males | Anxiety | Anger | -- | -- | -- | -- | -- |
|  | 53 | Males | Health & Functioning | Anger | -- | -- | -- | -- | -- |
|  | 53 | Males | PTSD | Anger | -- | -- | -- | -- | -- |
|  | 53 | Males | Depression | Anger | -- | -- | -- | -- | -- |
|  | 53 | Males | Health & Functioning | Anger | -- | -- | -- | -- | -- |
| Mohr et al  (2011) | 56 | Males | Depression | Depression | Phone | 20 | Facilitated | No | 22 |
|  | 56 | Males | Depression | Depression | Phone | 20 | Facilitated | No | 22 |
|  | 56 | Males | Depression | Depression | -- | -- | -- | -- | -- |
|  | 56 | Males | Depression | Depression | -- | -- | -- | -- | -- |
| Nelson et al  (2014) | 50 | Mixed | Depression | Depression | Computer | 8 | Facilitated | Yes | 37 |
|  | 50 | Mixed | Anxiety | Depression | Computer | 8 | Facilitated | Yes | 37 |
|  | 50 | Mixed | Health & Functioning | Depression | Computer | 8 | Facilitated | Yes | 37 |
| Pfeiffer et al  (2020) | 52 | Mixed | Health & Functioning | Depression | Computer | 12 | Facilitated | No | 28 |
|  | 52 | Mixed | Health & Functioning | Depression | Computer | 12 | Facilitated | No | 28 |
|  | 52 | Mixed | Health & Functioning | Depression | Computer | 12 | Facilitated | No | 28 |
|  | 52 | Mixed | Health & Functioning | Depression | Computer | 12 | Facilitated | No | 28 |
|  | 52 | Mixed | Health & Functioning | Depression | -- | -- | -- | -- | -- |
|  | 52 | Mixed | Health & Functioning | Depression | -- | -- | -- | -- | -- |
|  | 52 | Mixed | Health & Functioning | Depression | -- | -- | -- | -- | -- |
|  | 52 | Mixed | Health & Functioning | Depression | -- | -- | -- | -- | -- |
| Possemato et al  (2015) | 42 | Males | PTSD | PTSD | Mobile App | 8 | Facilitated | No | 0 |
|  | 42 | Males | Depression | PTSD | Mobile App | 8 | Facilitated | No | 0 |
|  | 42 | Males | QoL | PTSD | Mobile App | 8 | Facilitated | No | 0 |
|  | 42 | Males | QoL | PTSD | Mobile App | 8 | Facilitated | No | 0 |
|  | 42 | Males | PTSD | PTSD | -- | -- | -- | -- | -- |
|  | 42 | Males | Depression | PTSD | -- | -- | -- | -- | -- |
|  | 42 | Males | QoL | PTSD | -- | -- | -- | -- | -- |
|  | 42 | Males | QoL | PTSD | -- | -- | -- | -- | -- |
| Possemato et al  (2019) | 39 | Males | PTSD | Substance Use | Web | 12 | Facilitated | Yes | 27 |
|  | 39 | Males | Substance Use | Substance Use | Web | 12 | Facilitated | Yes | 27 |
|  | 39 | Males | Substance Use | Substance Use | Web | 12 | Facilitated | Yes | 27 |
|  | 39 | Males | QoL | Substance Use | Web | 12 | Facilitated | Yes | 27 |
|  | 39 | Males | QoL | Substance Use | Web | 12 | Facilitated | Yes | 27 |
|  | 39 | Males | Health & Functioning | Substance Use | Web | 12 | Facilitated | Yes | 27 |
|  | 39 | Males | Health & Functioning | Substance Use | Web | 12 | Facilitated | Yes | 27 |
|  | 39 | Males | Depression | Substance Use | Web | 12 | Facilitated | Yes | 27 |
|  | 39 | Males | PTSD | Substance Use | -- | -- | -- | -- | -- |
|  | 39 | Males | Substance Use | Substance Use | -- | -- | -- | -- | -- |
|  | 39 | Males | Substance Use | Substance Use | -- | -- | -- | -- | -- |
|  | 39 | Males | QoL | Substance Use | -- | -- | -- | -- | -- |
|  | 39 | Males | QoL | Substance Use | -- | -- | -- | -- | -- |
|  | 39 | Males | Health & Functioning | Substance Use | -- | -- | -- | -- | -- |
|  | 39 | Males | Health & Functioning | Substance Use | -- | -- | -- | -- | -- |
|  | 39 | Males | Depression | Substance Use | -- | -- | -- | -- | -- |
| Pulantara et al  (2018) | 36 | Mixed | Behavior | Sleep | Mobile App | 6 | Self-Guided | Yes | 18 |
|  | 36 | Mixed | Behavior | Sleep | Mobile App | 6 | Self-Guided | Yes | 18 |
|  | 36 | Mixed | Behavior | Sleep | Mobile App | 6 | Self-Guided | Yes | 18 |
|  | 36 | Mixed | Behavior | Sleep | Mobile App | 6 | Self-Guided | Yes | 18 |
|  | 36 | Mixed | PTSD | Sleep | Mobile App | 6 | Self-Guided | Yes | 18 |
|  | 36 | Mixed | Depression | Sleep | Mobile App | 6 | Self-Guided | Yes | 18 |
|  | 36 | Mixed | Anxiety | Sleep | Mobile App | 6 | Self-Guided | Yes | 18 |
| Stecker et al  (2014) | 28 | Mixed | PTSD | PTSD | Phone | 1 | Facilitated | Yes | 0 |
|  | 28 | Mixed | Depression | PTSD | Phone | 1 | Facilitated | Yes | 0 |
|  | 28 | Mixed | PTSD | PTSD | -- | -- | -- | -- | -- |
|  | 28 | Mixed | Depression | PTSD | -- | -- | -- | -- | -- |
| Taylor et al  (2017) | 33 | Mixed | Behavior | Sleep | Web | 6 | Self-Guided | Yes | 21 |
|  | 33 | Mixed | Behavior | Sleep | Web | 6 | Self-Guided | Yes | 21 |
|  | 33 | Mixed | Behavior | Sleep | Web | 6 | Self-Guided | Yes | 21 |
|  | 33 | Mixed | Behavior | Sleep | Web | 6 | Self-Guided | Yes | 21 |
|  | 33 | Mixed | Behavior | Sleep | Web | 6 | Self-Guided | Yes | 21 |
|  | 33 | Mixed | Behavior | Sleep | Web | 6 | Self-Guided | Yes | 21 |
|  | 33 | Mixed | Behavior | Sleep | -- | -- | -- | -- | -- |
|  | 33 | Mixed | Behavior | Sleep | -- | -- | -- | -- | -- |
|  | 33 | Mixed | Behavior | Sleep | -- | -- | -- | -- | -- |
|  | 33 | Mixed | Behavior | Sleep | -- | -- | -- | -- | -- |
|  | 33 | Mixed | Behavior | Sleep | -- | -- | -- | -- | -- |
|  | 33 | Mixed | Behavior | Sleep | -- | -- | -- | -- | -- |
|  | 33 | Mixed | Behavior | Sleep | -- | -- | -- | -- | -- |
|  | 33 | Mixed | Behavior | Sleep | -- | -- | -- | -- | -- |
|  | 33 | Mixed | Behavior | Sleep | -- | -- | -- | -- | -- |
|  | 33 | Mixed | Behavior | Sleep | -- | -- | -- | -- | -- |
|  | 33 | Mixed | Behavior | Sleep | -- | -- | -- | -- | -- |
|  | 33 | Mixed | Behavior | Sleep | -- | -- | -- | -- | -- |
| Timmons  (1997) | n/a | Males | Anxiety | Anger | Computer | 3 | Self-Guided | Yes | 13 |
|  | n/a | Males | Anxiety | Anger | Computer | 3 | Self-Guided | Yes | 13 |
|  | n/a | Males | Anxiety | Anger | -- | -- | -- | -- | -- |
|  | n/a | Males | Anxiety | Anger | -- | -- | -- | -- | -- |
|  | n/a | Males | Anxiety | Anger | -- | -- | -- | -- | -- |
|  | n/a | Males | Anxiety | Anger | -- | -- | -- | -- | -- |
| Voorhees et al  (2012) | 30 | Mixed | Depression | PTSD | Web | 12 | Self-Guided | Yes | 18 |
|  | 30 | Mixed | PTSD | PTSD | Web | 12 | Self-Guided | Yes | 18 |
|  | 30 | Mixed | QoL | PTSD | Web | 12 | Self-Guided | Yes | 18 |

*Notes*. PTSD = post traumatic stress disorder; QoL = quality of life; mTBI = mild traumatic brain injury; n/a = not available

*Table S1.* *Included Measures from Each Study*

| **Study** | **Outcome Measured** | **Target Outcome** | **Measures**  **Used** |
| --- | --- | --- | --- |
| Acosta et al (2017) | Substance Use | Primary | AUDIT |
|  | PTSD | Primary | CAPS |
|  | QoL | Secondary | WHOQOL-BREF |
| Belsher et al (2015 | PTSD | Primary | PCL-M |
| Cooper et al (2017) | Health & Functioning | Primary | SCL-90-R |
| Dobkin et al (2020) | Anxiety | Secondary | HAMA |
|  | Behavior | Secondary | ATQ |
|  | Health & Functioning | Secondary | CGI-I |
|  | QoL | Secondary | SF-36 |
|  | Depression | Primary | HAMD |
| Engel et al (2015) | PTSD | Primary | PCL-C |
|  | Depression | Secondary | PHQ-8 |
| Engel et al (2021) | PTSD | Primary | CAPS-5 |
| Herbst et al (2018) | Substance Use | Primary | Fagerström Test for Nicotine Dependence |
|  | Health & Functioning | Secondary | DSM-5 |
| Hobfoll et al (2016) | PTSD | Primary | PCL-M |
|  | Depression | Secondary | CES-D-10 |
| Litz et al  (2007) | Anxiety | Secondary | BAI |
|  | Depression | Secondary | BDI-II |
|  | PTSD | Primary | PCL (modified) |
| Mackintosh et al (2017) | PTSD | Primary | PCL-5 |
|  | Depression | Secondary | PHQ-9 |
|  | Health & Functioning | Secondary | B-IPF |
|  | Anxiety | Primary | STAXI-AEI |
|  | Health & Functioning | Secondary | DAR-5 |
| Mohr et al (2011) | Depression | Primary | HAM-D |
| Nelson et al (2014) | Depression | Primary | BDI-II |
|  | Anxiety | Secondary | BAI |
|  | Health & Functioning | Secondary | SHS |
| Pfeiffer et al (2020) | Depression | Primary | QIDS-SR |
|  | Health & Functioning | Secondary | VR-12 MCS |
|  | QoL | Secondary | QLES-Q-SF |
| Possemato et al (2016) | PTSD | Primary | PCL |
|  | Depression | Secondary | PHQ-9 |
|  | QoL | Secondary | QHO-QoL |
| Possemato et al (2019)a | PTSD | Primary | PCL-M |
|  | Substance Use | Secondary | Drinking days |
|  | QoL | Secondary | QHO-QoL |
|  | Health & Functioning | Secondary | CSC |
|  | Depression | Primary | PAM-MH |
| Possemato et al (2019)b | PTSD | Primary | PCL-M |
|  | Substance Use | Secondary | Drinking days |
|  | QoL | Secondary | QHO-QoL |
|  | Health & Functioning | Secondary | CSC |
|  | Depression | Primary | PAM-MH |
| Pulantara et al (2018) | Behavior | Primary | ISI |
|  | PTSD | Secondary | PCL-C |
|  | Depression | Secondary | PHQ-9 |
|  | Anxiety | Secondary | GAD-7 |
| Stecker et al (2014) | PTSD | Primary | PCL |
|  | Depression | Secondary | PHQ-9 |
| Taylor et al (2017) | Behavior | Primary | Sleep Efficiency |
| Timmons (1997) | Anxiety | Primary | STAXI |
| Voorhees et al (2012) | Depression | Secondary | CES-D |
|  | PTSD | Primary | PCL-M |
|  | QoL | Secondary | SF-12 |

**Articles include in meta-analysis**

1. Acosta, M. C., Possemato, K., Maisto, S. A., Marsch, L. A., Barrie, K., Lantinga, L., Fong, C., Xie, H., Grabinski, M., & Rosenblum, A. (2017). Web-Delivered CBT Reduces Heavy Drinking in OEF-OIF Veterans in Primary Care With Symptomatic Substance Use and PTSD. *Behav Ther*, *48*(2), 262-276. https://doi.org/10.1016/j.beth.2016.09.001
2. Belsher, B. E., Kuhn, E., Maron, D., Prins, A., Cueva, D., Fast, E., & France, D. (2015). A preliminary study of an internet-based intervention for OEF/OIF veterans presenting for VA specialty PTSD care. *J Trauma Stress*, *28*(2), 153-156. https://doi.org/10.1002/jts.21994
3. Cooper, D. B., Bowles, A. O., Kennedy, J. E., Curtiss, G., French, L. M., Tate, D. F., & Vanderploeg, R. D. (2017). Cognitive Rehabilitation for Military Service Members With Mild Traumatic Brain Injury: A Randomized Clinical Trial. *J Head Trauma Rehabil*, *32*(3), E1-E15. https://doi.org/10.1097/HTR.0000000000000254
4. Dobkin, R. D., Mann, S. L., Weintraub, D., Rodriguez, K. M., Miller, R. B., St Hill, L., King, A., Gara, M. A., & Interian, A. (2021). Innovating Parkinson’s Care: A Randomized Controlled Trial of Telemedicine Depression Treatment. *Mov Disord*, *36*(11), 2549-2558. https://doi.org/10.1002/mds.28548
5. Engel, C. C., Litz, B., Magruder, K. M., Harper, E., Gore, K., Stein, N., Yeager, D., Liu, X., & Coe, T. R. (2015). Delivery of self training and education for stressful situations (DESTRESS-PC): a randomized trial of nurse assisted online self-management for PTSD in primary care. *Gen Hosp Psychiatry*, *37*(4), 323-328. https://doi.org/10.1016/j.genhosppsych.2015.04.007
6. Engel, S., Schumacher, S., Niemeyer, H., Kuester, A., Burchert, S., Klusmann, H., Rau, H., Willmund, G. D., & Knaevelsrud, C. (2021). Associations between oxytocin and vasopressin concentrations, traumatic event exposure and posttraumatic stress disorder symptoms: group comparisons, correlations, and courses during an internet-based cognitive-behavioural treatment. *Eur J Psychotraumatol*, *12*(1), 1886499. https://doi.org/10.1080/20008198.2021.1886499
7. Herbst, E., Pennington, D., Kuhn, E., McCaslin, S. E., Delucchi, K., Batki, S. L., Dickter, B., & Carmody, T. (2018). Mobile Technology for Treatment Augmentation in Veteran Smokers With Posttraumatic Stress Disorder. *Am J Prev Med*, *54*(1), 124-128. https://doi.org/10.1016/j.amepre.2017.08.016
8. Hobfoll, S. E., Blais, R. K., Stevens, N. R., Walt, L., & Gengler, R. (2016). Vets prevail online intervention reduces PTSD and depression in veterans with mild-to-moderate symptoms. *J Consult Clin Psychol*, *84*(1), 31-42. https://doi.org/10.1037/ccp0000041
9. Litz, B. T., Engel, C. C., Bryant, R. A., & Papa, A. (2007). A randomized, controlled proof-of-concept trial of an Internet-based, therapist-assisted self-management treatment for posttraumatic stress disorder. *Am J Psychiatry*, *164*(11), 1676-1683. https://doi.org/10.1176/appi.ajp.2007.06122057
10. Mackintosh, M. A., Niehaus, J., Taft, C. T., Marx, B. P., Grubbs, K., & Morland, L. A. (2017). Using a Mobile Application in the Treatment of Dysregulated Anger Among Veterans. *Mil Med*, *182*(11), e1941-e1949. https://doi.org/10.7205/MILMED-D-17-00063
11. Mohr, D. C., Carmody, T., Erickson, L., Jin, L., & Leader, J. (2011). Telephone-administered cognitive behavioral therapy for veterans served by community-based outpatient clinics. *J Consult Clin Psychol*, *79*(2), 261-265. https://doi.org/10.1037/a0022395
12. Nelson, C. B., Abraham, K. M., Walters, H., Pfeiffer, P. N., & Valenstein, M. (2014). Integration of peer support and computer-based CBT for veterans with depression. *Computers in Human Behavior*, *31*, 57-64. https://doi.org/10.1016/j.chb.2013.10.012
13. Pfeiffer, P. N., Pope, B., Houck, M., Benn-Burton, W., Zivin, K., Ganoczy, D., Kim, H. M., Walters, H., Emerson, L., Nelson, C. B., Abraham, K. M., & Valenstein, M. (2020). Effectiveness of Peer-Supported Computer-Based CBT for Depression Among Veterans in Primary Care. *Psychiatr Serv*, *71*(3), 256-262. https://doi.org/10.1176/appi.ps.201900283
14. Possemato, K., Kuhn, E., Johnson, E., Hoffman, J. E., Owen, J. E., Kanuri, N., De Stefano, L., & Brooks, E. (2016). Using PTSD Coach in primary care with and without clinician support: a pilot randomized controlled trial. *Gen Hosp Psychiatry*, *38*, 94-98. https://doi.org/10.1016/j.genhosppsych.2015.09.005
15. Possemato, K., Johnson, E. M., Emery, J. B., Wade, M., Acosta, M. C., Marsch, L. A., Rosenblum, A., & Maisto, S. A. (2019). A pilot study comparing peer supported web-based CBT to self-managed web CBT for primary care veterans with PTSD and hazardous alcohol use. *Psychiatr Rehabil J*, *42*(3), 305-313. https://doi.org/10.1037/prj0000334
16. Pulantara, I. W., Parmanto, B., & Germain, A. (2018). Clinical Feasibility of a Just-in-Time Adaptive Intervention App (iREST) as a Behavioral Sleep Treatment in a Military Population: Feasibility Comparative Effectiveness Study. *J Med Internet Res*, *20*(12), e10124. https://doi.org/10.2196/10124
17. Stecker, T., McHugo, G., Xie, H., Whyman, K., & Jones, M. (2014). RCT of a brief phone-based CBT intervention to improve PTSD treatment utilization by returning service members. *Psychiatr Serv*, *65*(10), 1232-1237. https://doi.org/10.1176/appi.ps.201300433
18. Taylor, D. J., Peterson, A. L., Pruiksma, K. E., Young-McCaughan, S., Nicholson, K., Mintz, J., & STRONG, S. T. A. R. C. (2017). Internet and In-Person Cognitive Behavioral Therapy for Insomnia in Military Personnel: A Randomized Clinical Trial. *Sleep*, *40*(6). https://doi.org/10.1093/sleep/zsx075
19. Timmons, P. L., Oehlert, M. E., Sumerall, S. W., Timmons, C. W., & Borgers, S. B. (1997). Stress inoculation training for maladaptive anger: Comparison of group counseling versus computer guidance. *Computers in Human Behavior*, *13*(1), 51-64. <https://doi.org/10.1016/s0747-5632(96)00029-5>
20. Van Voorhees, B. W., Gollan, J., & Fogel, J. (2012). Pilot study of Internet-based early intervention for combat-related mental distress. *J Rehabil Res Dev*, *49*(8), 1175-1190. https://doi.org/10.1682/jrrd.2011.05.0095
